# Fluctuating Regional Brainstem Diffusion Imaging Measures of Microstructure across the Migraine Cycle

**DOI:** 10.1523/ENEURO.0005-19.2019

**Published:** 2019-07-25

**Authors:** Kasia K. Marciszewski, Noemi Meylakh, Flavia Di Pietro, Vaughan G. Macefield, Paul M. Macey, Luke A. Henderson

**Affiliations:** 1 Department of Anatomy and Histology, Sydney Medical School, University of Sydney, Sydney, 2006 New South Wales, Australia; 2 School of Medicine, Western Sydney University, Campbelltown, 2560 New South Wales, Australia; 3 University of California, Los Angeles School of Nursing and Brain Research Institute, University of California, Los Angeles, CA 90095

**Keywords:** brainstem, diffusion tensor imaging, migraine, MRI, PAG, spinal trigeminal nucleus

## Abstract

The neural mechanisms responsible for the initiation and expression of migraines remain unknown. Although there is growing evidence of changes in brainstem anatomy and function between attacks, very little is known about brainstem function and structure in the period immediately prior to a migraine. The aim of this investigation is to use brainstem-specific analyses of diffusion weighted images to determine whether the brainstem pain processing regions display altered structure in individuals with migraine across the migraine cycle, and in particular immediately prior to a migraine. Diffusion tensor images (29 controls, 36 migraineurs) were used to assess brainstem anatomy in migraineurs compared with controls. We found that during the interictal phase, migraineurs displayed greater mean diffusivity (MD) in the region of the spinal trigeminal nucleus (SpV), dorsomedial pons (dmPons)/dorsolateral pons (dlPons), and midbrain periaqueductal gray matter (PAG)/cuneiform nucleus (CNF). Remarkably, the MD returned to controls levels during the 24-h period immediately prior to a migraine, only to increase again within the three following days. Additionally, fractional anisotropy (FA) was significantly elevated in the region of the medial lemniscus/ventral trigeminal thalamic tract in migraineurs compared with controls over the entire migraine cycle. These data show that regional brainstem anatomy changes over the migraine cycle, with specific anatomical changes occurring in the 24-h period prior to onset. These changes may contribute to the activation of the ascending trigeminal pathway by either an increase in basal traffic or by sensitizing the trigeminal nuclei to external triggers, with activation ultimately resulting in perception of head pain during a migraine attack.

## Significance Statement

It has been hypothesized that modulation of brainstem pain pathways may be critical for the initiation of migraine attacks. There is some evidence that altered brainstem function, possibly involving increased astrocyte activation, occurs immediately before a migraine attack. We sought to obtain evidence to support this theory. Using diffusion tensor imaging (DTI), we found that immediately before a migraine, mean diffusivity (MD) decreased in the spinal trigeminal nucleus (SpV), dorsomedial pons (dmPons)/dorsolateral pons (dlPons), and midbrain periaqueductal gray matter (PAG)/nucleus cuneiform. MD then increased again immediately following the migraine attack. Decreased MD before a migraine is consistent with increased astrocyte activation, since astrocyte processes enlarge during activation. These changes may underlie changes in brainstem function that are essential for the generation of a migraine.

## Introduction

Migraine is a common, debilitating disorder characterized by headaches and often accompanied by aura, nausea, and sensitivity to light and sound. Despite these well-characterized symptoms, the exact mechanisms underlying the initiation and maintenance of migraine head pain are still hotly debated. To date, human brain imaging investigations have revealed that during a migraine attack, activity increases in brain regions such as the cingulate cortex, insula, thalamus, hypothalamus and dorsal pons (Weiller et al., 1995; Bahra et al., 2001; Denuelle et al., 2004; Afridi et al., 2005). In addition, a number of studies have identified anatomical, sensitivity, and resting activity pattern changes between migraine attacks, i.e., in the interictal period (Chong and Schwedt, 2015; Mathur et al., 2016; Chong et al., 2017; Porcaro et al., 2017; Marciszewski et al., 2018a). These findings highlight the apparent brain dysfunction in migraineurs even when in a pain-free state.

A recent review has proposed that these observed changes in brain function are not stable but dynamic in nature (May, 2017). Some have suggested that functional brain changes actually trigger a migraine from basal firing (Goadsby and Akerman, 2012). Others have suggested that the brain fluctuates between a state where the effectiveness of endogenous analgesic mechanisms is too great to allow incoming noxious inputs to evoke head pain, and a state where incoming inputs can activate central pathways and evoke head pain (Akerman et al., 2011; Borsook and Burstein, 2012). Consistent with these hypotheses, it has recently been reported that during the interictal phase, migraineurs display reduced gray matter density and increased free water movement within brainstem pain-modulating regions including the midbrain periaqueductal gray matter (PAG), dorsolateral pons (dlPons), medullary raphe and spinal trigeminal nucleus (SpV; Marciszewski et al., 2018a). Furthermore, it was recently shown that immediately before a migraine resting infra-slow oscillatory activity increases in these same brainstem regions and returns to controls levels shortly after the migraine and it was speculated that these oscillatory changes may result from transient increases in astrocyte activation and its associated gliotransmission (Meylakh et al., 2018). Given there is some evidence that astrocytes may play a role in aspects of migraine such as the propagation of cortical spreading depression (Nedergaard et al., 1995) and that a genetic form of migraine, familial hemiplegic migraine, is associated with astrocyte dysfunction (Benarroch, 2005), it is not unreasonable to suggest that astrocytes may also play a critical role in migraine pathophysiology via actions within the brainstem.

It was recently reported that pain sensitivity to noxious stimuli in migraineurs is dramatically decreased in the 24-h period before a migraine, and this decrease is associated with increased functional magnetic resonance imaging (fMRI) signal intensity within the SpV and reduced PAG-SpV connectivity (Marciszewski et al., 2018b). This altered brainstem function may result from altered neural-glial interactions, although evidence of astrocyte activation in migraineurs, particularly in the period immediately before a migraine is lacking. This is likely due to the fact that it is not possible to predict when an individual will have a migraine and thus examining them in the 24-h period before an attack is exceptionally difficult. Local free water movement, as measured by diffusion tensor imaging (DTI), is a method by which microstructural alterations can be examined in living humans. Such diffusion measures can be altered by numerous processes that change local anatomy, including edema, inflammation, demyelination and alterations in cell numbers and/or morphology. Furthermore, astrocyte activation, which is associated with their processes enlarging considerably, would result in a decrease in local free water movement.

The aim of this investigation is to use DTI to determine whether the brainstem displays microstructural alterations throughout the migraine cycle. More specifically we aim to explore changes in the 24-h period before a migraine. We hypothesize that immediately before a migraine, mean diffusivity (MD) will decrease, consistent with an increase in astrocyte size, in areas of the brainstem that process and modulate pain such the PAG, dlPons, medullary raphe, and SpV. Furthermore, that decrease will be reversed during the period immediately following a migraine and return to interictal levels.

## Materials and Methods

### Subjects

Thirty-six subjects with migraine (eight males, mean ± SEM age: 30.6 ± 1.7 years) and 29 age and gender matched pain-free controls (five males, age: 31.9 ± 2.3 years) were recruited for the study. All subjects were recruited from the general population using an advertisement. Migraine subjects were diagnosed according to the criteria laid out by the International Classification of Headache Disorders (ICHD), 3rd edition, sections 1.1 and 1.2 (ICHD-3β, 2013). Seven migraineurs reported aura associated with their migraines and the remaining 29 reported no aura. Of the 36 migraineurs, 31 were scanned during the interictal period (seven males, age 30.1 ± 1.9 years), that is, between 72 h after and 24 h before a migraine attack; 13 during the 24-h period immediately before a migraine (four males, age 26.0 ± 2.4 years), and 15 within the 72-h period following a migraine (four males, age 31.5 ± 2.5 years). For subjects scanned before an attack, there was no predicting factor that they were within 24 h of a migraine. Eleven migraineurs were scanned during the interictal period and period immediately before a migraine. In addition, seven of these 10 subjects were also scanned during the period immediately after a migraine.

All migraine subjects indicated the pain intensity (six-point visual analog scale; 0 = no pain, 5 = most intense imaginable pain) and facial distribution (drawing) of pain they commonly experience during a migraine attack. Each subject described the qualities of their migraines and indicated any current treatments used to prevent or abort a migraine once started. Exclusion criteria for controls were the presence of any current pain or chronic pain condition, current use of analgesics, and any neurological disorder. Exclusion criteria for migraineurs were any pain condition other than migraine, and any other neurological disorder. Informed written consent was obtained for all procedures according to the Declaration of Helsinki 7th revision and local Institutional Human Research Ethics Committees approved the study.

### MRI acquisition

Subjects lay supine on the bed of a 3T MRI scanner (Philips Achieva, Neuroscience Research Australia) with their head immobilized in a fitting 32-channel head coil. With all subjects relaxed and at rest, in each subject a high-resolution T1-weighted anatomical image set covering the entire brain was collected (turbo field echo; field of view 250 × 250 mm, matrix size = 288 × 288, slice thickness = 0.87 mm, repetition time = 5600 ms, echo time = 2.5 ms, flip angle 8°). Following this, two high-resolution DTI image sets covering the entire brain were collected using a single-shot multisection spin-echo echo-planar pulse sequence (repetition time = 8788 ms; flip angle = 90°, matrix size 112 × 112, field of view 224 × 224 mm, slice thickness = 2.5 mm, 55 axial slices). For each slice, diffusion gradients were applied along 32 independent orientations with *b* = 1000 s/mm^2^ after the acquisition of five *b* = 0 s/mm^2^ (b0) images. Two DTI acquisitions were averaged to improve signal-noise ratios.

### Image analysis

#### DTI analysis

Using SPM12 software (Friston et al., 1994), the two DTI image sets from each subject were realigned based on the b0 images, and the diffusion tensors calculated from the images using a linear model (Basser and Pierpaoli, 1996). MD, axial diffusivity, radial diffusivity, and fractional anisotropy (FA) whole-brain maps were then derived and co-registered to each individual subject’s T1-weighted image. Using brainstem-specific isolation software (SUIT toolbox; Diedrichsen, 2006), a unique mask of the brainstem was manually created for each subject’s MD map. Using these masks, the brainstem was isolated from the MD, axial diffusivity, radial diffusivity and FA maps, spatially normalized, re-sliced to the SUIT template in Montreal Neurologic Institute (MNI) space, and spatially smoothed using a 3-mm full-width-at-half-maximum (FWHM) Gaussian filter.

#### Statistical analyses

Using a voxel-by-voxel analysis, significant differences in MD and FA values were determined between (1) controls (*n* = 29) and migraineurs during the interictal period (*n* = 31), (2) controls and migraineurs during the period immediately *before* a migraine (*n* = 13), and (3) controls and migraineurs during the period immediately following a migraine (*n* = 15; all comparisons *p* < 0.05, false discovery rate corrected at a voxel level, minimum cluster size 5 contiguous voxels). Age and gender were included as nuisance variables and a brainstem mask that excluded cerebrospinal fluid as well as the cerebellum was applied to each analysis.

Since we found significant MD increases during the interictal period that were eliminated immediately before a migraine, we extracted MD values from those significant clusters for all three migraine periods. Significant MD differences between controls and the period immediately before and following migraine were then determined for these clusters (two-tailed, two-sample *t* test, *p* < 0.05). In addition, the axial diffusivity, radial diffusivity and FA values from each of the significant clusters were extracted and plotted and significant differences between controls and each migraine period as well as between migraine periods themselves were determined (two-tailed, two-sample *t* test, *p* < 0.05). We also found a cluster of significant FA change that was present during all three migraine phases. We extracted the MD, axial diffusivity, radial diffusivity, and FA values from significant clusters of the control versus interictal analysis for all three migraine periods and determined significance relative to controls and between migraine phases (two-tailed, two-sample *t* test, *p* < 0.05). Significant MD and FA differences between controls and the interictal period were not assessed a second time since these were already established as significant with the voxel-based statistics, thus avoiding the issue of “double-dipping.”

To explore changes in individual migraineurs throughout the migraine cycle, in 11 migraineurs that were scanned during more than one period, we plotted MD and FA values for each significant cluster identified in the original group analysis. Significant MD and FA differences between each of the three migraine periods were then determined using paired *t* tests (two-tailed *p* < 0.05). Additionally, for all migraineurs, MD and FA values relative to the time until next migraine were plotted and a line of best fit applied for each significant cluster determined in the original group analysis to explore whether MD and FA increased or decreased as a migraine event approached. Finally, for each cluster, significant relationships between MD and FA and migraine characteristics were determined (Pearson correlation, *p* < 0.05).

## Results

### Migraine characteristics

Using a self-report questionnaire, migraineurs reported the most common location of their migraines over the past 12 months. In 12 of the 36 migraineurs, headaches were more common on the right side, in six they were more common on the left, and in the remaining 18 they were most often bilateral. Migraine subjects most frequently described their migraine pain as “throbbing,” “pulsating,” and/or “sharp” in nature. They indicated that “stress,” “lack of sleep,” and “dehydration” most often triggered migraine attacks. The mean (±SEM) estimated frequency of migraine attacks was 1.3 ± 0.1 per month, mean length of time since the onset of migraine attacks (years suffering) 16.2 ± 1.9 years, and mean pain intensity of migraines was 3.8 ± 0.1 on a six-point visual analog scale. Although 24 of the 36 migraineurs were taking some form of daily medication (mostly the oral contraceptive pill), none of the migraine subjects were taking prophylactic medication prescribed for migraine.

### Diffusivity measures

#### Group comparisons

**Figure 1.**
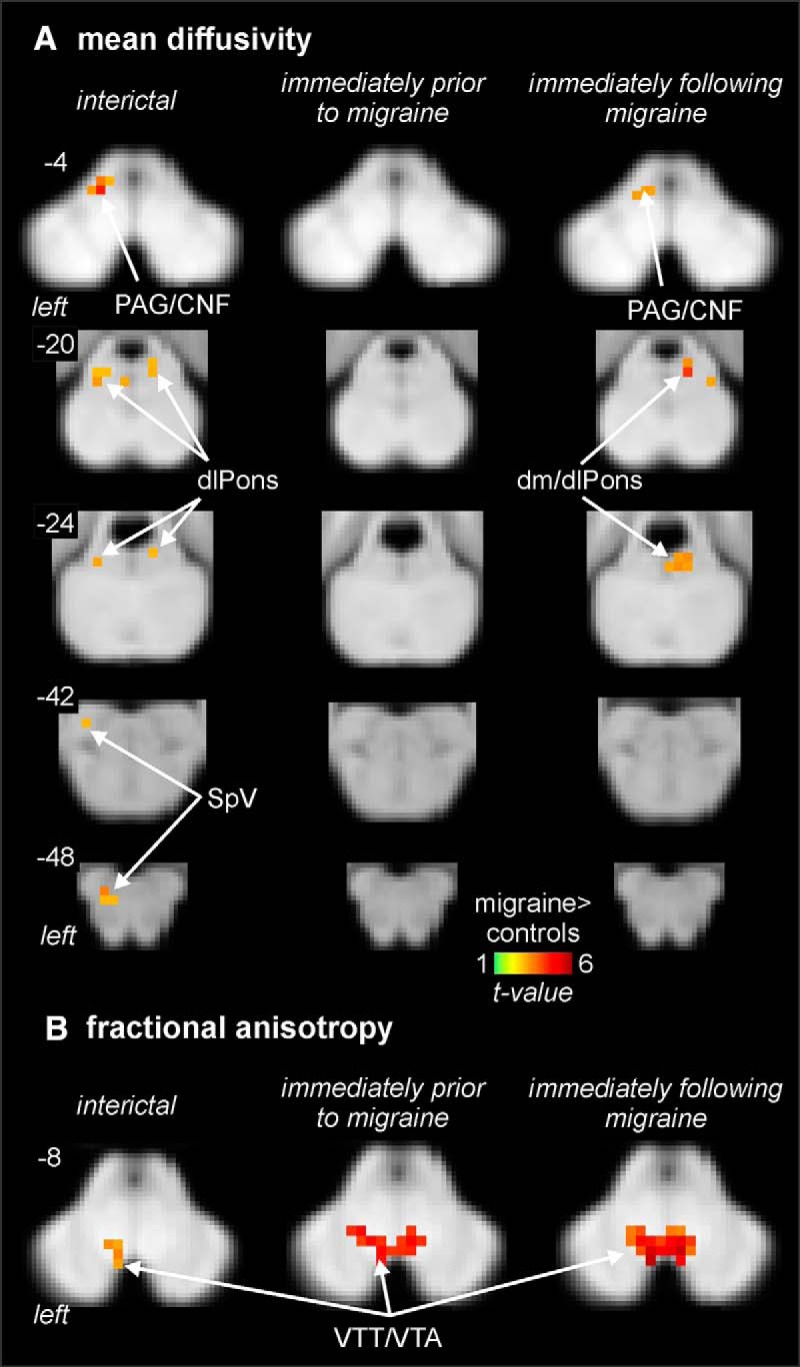
Regional MD (***A***) and FA differences (***B***) in migraineurs during the interictal (*n* = 31), immediately before migraine (*n* = 13), and immediately following a migraine (*n* = 15) compared with controls (*n* = 29). Significantly different clusters are overlaid onto axial brainstem template images and significant increases in migraineurs are represented by a *t* value with a hot color scale. Slice locations are indicated at the upper left of each axial slice in MNI space. Compared to controls, migraineurs have increased MD during the interictal and immediately following migraine periods in the region of the left SpV, left and right dlPons, and in the region encompassing the PAG/CNF. In addition, migraineurs display an increase in FA in the region of the VTT/VTA during all three phases compared with controls.

**Figure 2.**
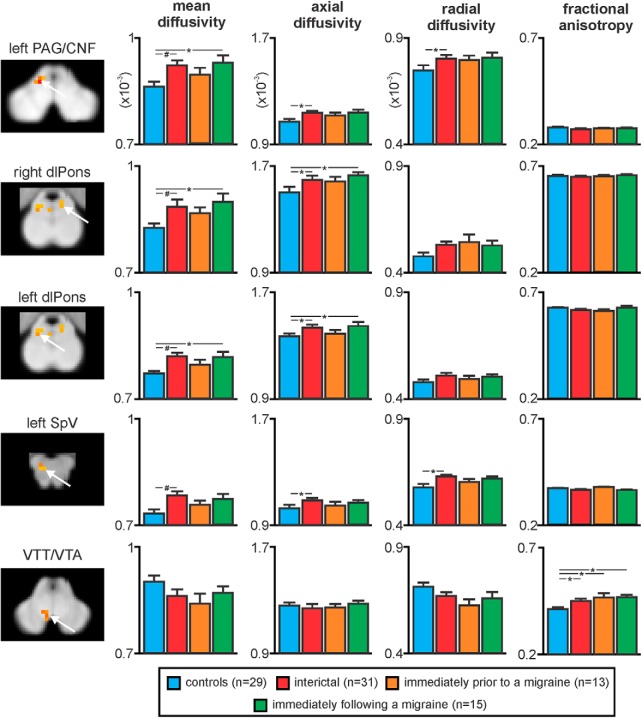
Plots of mean (±SEM) MD, axial diffusivity, radial diffusivity, and FA values in migraineurs compared with pain-free controls in the left SpV, left and right dlPons, left PAG/CNF, and in the VTT/VTA. Consistent with the voxel-by-voxel analysis, MD was significantly increased during the interictal and immediately following a migraine phase but not immediately before a migraine. In addition, axial diffusivity showed a largely similar pattern of difference in migraineurs whereas radial diffusion was only different during the interictal phase in the PAG/CNF and SpV. In contrast, FA was significantly increased in the area of the VTT/VTA in migraineurs during all three phases; #*p* < 0.05 voxel-by-voxel analysis; *p < 0.05 two-sample *t* tests.

The DTI analysis revealed that compared to controls, migraineurs show regional differences in brainstem MD throughout the migraine cycle (Fig. 1; Table 1). Consistent with our previous report, during the interictal period migraineurs showed increased MD compared with controls in regions encompassing the left SpV, left dlPons, right dorsomedial pons (dmPons)/dlPons, and the region of the midbrain PAG and including the region of the cuneiform nucleus (CNF; Fig. 1; Table 1). Strikingly, this MD increase during the interictal period was absent during the 24-h period before a migraine, with no significant difference between controls and migraineurs in this period. The MD increase then returned to above control levels in the dm/dlPons and PAG/CNF in the 72-h period immediately following a migraine. Extraction of MD values from the clusters displaying a significant increase during the interictal period confirmed this pattern of MD change, i.e., MD increases during the interictal, no MD difference immediately before migraine and MD increase again immediately following a migraine (Fig. 2; Table 2). In no brainstem region was MD significantly lower in migraineurs compared with controls.

**Table 1.** MNI coordinates, cluster size, and *t* score for regions of significant increases in MD and FA in migraineurs compared with controls

Brain region	MNI coordinate			cluster size (voxels)	*t* score
	*x*	*y*	*z*		
Interictals>controls					
MD					
Left PAG/CNF Right dlPons Left dlPons Left SpV	–6 6 –4 –4	–28 –36 –32 –40	–3 –21 –13 –47	12 5 13 7	4.41 3.49 3.41 3.43
FA VTT/VTA	–2	–20	–9	10	3.74
Immediately prior to migraine>controls					
FA VTT/VTA	6	–20	–5	65	5.51
Immediately following a migraine>controls					
MD Left PAG/CNF Right dorsomedial/dlPons Left dlPons FA VTT/VTA	–6 6 –4 4	–30 –34 –32 –18	–4 –19 –23 –5	17 24 6 85	3.54 4.16 3.44 5.77

**Table 2.** Diffusion values for clusters displaying significant increases in MD or FA during the interictal phase in migraineurs compared with controls

Brain region	Diffusivity parameter	Controls	Interictal	Immediately prior to migraine	Immediately following migraine
Left PAG/CNF	MD	0.86 ± 0.01	0.92 ± 0.01	0.89 ± 0.01	0.93 ± 0.02
	Axial diffusivity	1.07 ± 0.02	1.14 ± 0.01	1.12 ± 0.02	1.14 ± 0.02
	Radial diffusivity	0.75 ± 0.01	0.81 ± 0.01	0.79 ± 0.02	0.81 ± 0.02
	FA	0.28 ± 0.01	0.27 ± 0.01	0.27 ± 0.01	0.27 ± 0.01
left dlPons	MD	0.77 ± 0.01	0.82 ± 0.01	0.79 ± 0.01	0.82 ± 0.01
	Axial diffusivity	1.37 ± 0.02	1.44 ± 0.01	1.40 ± 0.02	1.45 ± 0.02
	Radial diffusivity	0.47 ± 0.01	0.51 ± 0.01	0.49 ± 0.01	0.50 ± 0.02
	FA	0.63 ± 0.01	0.61 ± 0.01	0.61 ± 0.01	0.63 ± 0.01
right dl/dmPons	MD	0.83 ± 0.01	0.89 ± 0.1	0.86 ± 0.01	0.90 ± 0.01
	Axial diffusivity	1.52 ± 0.02	1.61 ± 0.02	1.58 ± 0.02	1.64 ± 0.02
	Radial diffusivity	0.49 ± 0.01	0.54 ± 0.02	0.55 ± 0.03	0.53 ± 0.02
	FA	0.66 ± 0.01	0.65 ± 0.01	0.65 ± 0.02	0.66 ± 0.01
SpV	MD	0.73 ± 0.01	0.79 ± 0.01	0.76 ± 0.1	0.78 ± 0.1
	Axial diffusivity	1.04 ± 0.02	1.10 ± 0.01	1.06 ± 0.01	1.08 ± 0.01
	Radial diffusivity	0.59 ± 0.01	0.63 ± 0.01	0.61 ± 0.01	0.62 ± 0.01
	FA	0.37 ± 0.01	0.36 ± 0.01	0.37 ± 0.01	0.36 ± 0.01
VTT/VTA	MD	0.90 ± 0.02	0.87 ± 0.02	0.84 ± 0.02	0.87 ± 0.01
	Axial diffusivity	1.26 ± 0.02	1.25 ± 0.02	1.25 ± 0.02	1.29 ± 0.02
	Radial diffusivity	0.72 ± 0.02	0.67 ± 0.02	0.64 ± 0.03	0.67 ± 0.01
	FA	0.41 ± 0.01	0.45 ± 0.01	0.47 ± 0.02	0.47 ± 0.01

MD, axial diffusivity, and radial diffusivity values are expressed as x10^−3^. The gray shaded boxes indicate a significant difference compared with controls.

Extraction of axial and radial diffusion values from these brainstem regions resulted in an interesting pattern of change (Table 2). Within the SpV, as with MD, both axial and radial diffusivity was significantly increased only during the interictal phase, and within the PAG/CNF axial and radial diffusivity was significantly increased during the interictal phase but unlike MD, it was not increased during the phase immediately following a migraine. Interestingly, within the left and right dlPons and dmPons regions, while axial diffusivity was significantly increased during the interictal and immediately following migraine phases, radial diffusivity was not significantly different during any migraine phase compared with controls and FA was not different during either phase or compared with controls.

Analysis of FA revealed a different pattern of change to that of the other diffusion measures. Migraineurs displayed a significant increase in FA in the area encompassing the ventral trigeminothalamic tract/ventral tegmental area (VTT/VTA) during all three phases (Fig. 1). Extraction of FA values from this region confirmed that FA was significantly increased in migraineurs and that this increase was apparent during all migraine phases (Fig. 2; Table 2).

#### Individual migraineur comparisons

**Figure 3.**
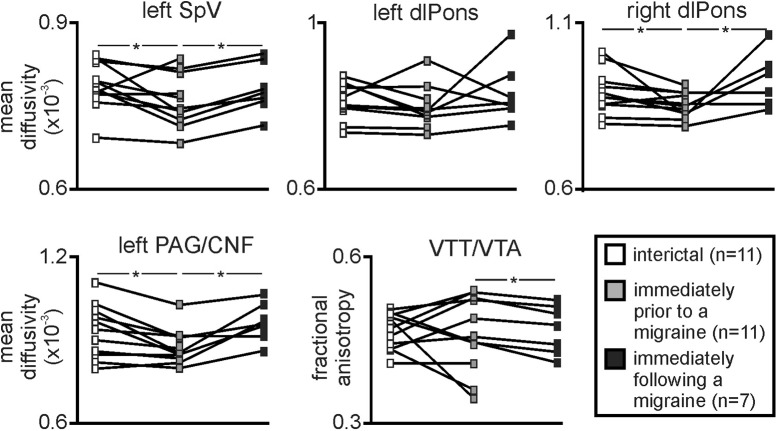
Plots of MD and FA in 11 migraineurs that were scanned during at least two of the three migraine phases. Note the consistency of MD change which decreases significantly during the period immediately before migraine in the left SpV, right dlPons, and left PAG/CNF. In contrast, FA within the area of the VTT/VTA was significantly greater during the period immediately before compared with immediately following a migraine; *p < 0.05 paired *t* tests.

Plots of MD values in the 11 migraineurs that were scanned during at least two of the three migraine periods revealed that the pattern of MD changes was consistent in individual subjects. That is, MD was lower immediately before a migraine compared with both the interictal and immediately following a migraine period (Fig. 3). While the left dlPons did not show a significant difference between phases, paired *t* tests revealed that the left SpV, right dlPons and left PAG/CNF displayed significantly reduced MD during the phase immediately before a migraine compared with both the interictal and immediately following a migraine phase. More specifically, of the 11 migraineurs, MD decreased immediately before a migraine compared to the interictal period in 10 migraineurs within the left SpV, left PAG/CNF, and right dm/dlPons and in nine migraineurs in the left dlPons. Additionally, of the seven migraineurs scanned during all three phases, all showed a MD decrease immediately before a migraine compared with both the interictal and immediately after a migraine within the left SpV, and five migraineurs for the left dlPons, right dm/dlPons, and PAG/CNF. In contrast to MD, consistent with the group analysis, FA was relatively consistent between the migraine phases, although it was significantly increased during the immediately before compared with the immediately following migraine phase.

**Figure 4.**
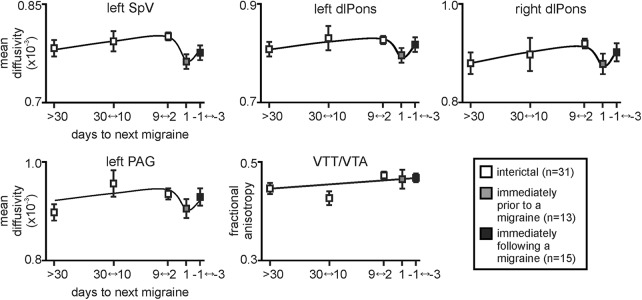
Plots of mean (±SEM) MD and FA changes in migraineurs over the migraine cycle in the left SpV, left and right dlPons, left PAG/CNF, and in the VTT/VTA. Values are averaged for those migraineurs scanned at least 30 d (*n* = 18), 30–10 d (*n* = 6), 9–2 d (*n* = 7), and 1 d before their next migraine (*n* = 13), as well as 1–3 d following a migraine (*n* = 15). Note how MD remains relatively stable over the interictal period and falls dramatically immediately before a migraine before recovering. In contrast, FA remains stable over all three migraine phases.

Furthermore, plots of MD and FA values over the migraine cycle revealed that within the SpV, right and left dlPons, and PAG/CNF, MD appeared to remain relatively stable across the interictal period before decreasing immediately before the migraine and then increasing again after the migraine (Fig. 4). In contrast, the elevated FA in the VTT/VTA remained relatively stable across the migraine cycle and did not change immediately before a migraine. Finally, in all migraine subject groups, MD or FA values in these clusters during the interictal phase were not significantly correlated to migraine frequency (MD: left SpV *r* = 0.03, left dlPons *r* = –0.13, right dm/dlPons *r* = 0.15, left PAG/CNF: *r* = 0.01; FA: VTT/VTA: *r* = 0.09; all *p* > 0.05), years suffering (MD: left SpV *r* = 0.11, left dlPons *r* = –0.09, right dm/dlPons *r* = –0.30, left PAG/CNF *r* = 0.14; FA: VTT/VTA *r* = 0.14; all *p* > 0.05), or the intensity of migraine pain (left SpV *r* = 0.31, left dlPons *r* = –0.11, right dm/dlPons *r* = –0.03, left PAG/CNF *r* = 0.09; FA: VTT/VTA *r* = –0.03; all *p* > 0.05).

## Discussion

This study demonstrates that migraine is associated with changes in regional anatomy that fluctuate over the migraine cycle in a number of brainstem regions. More specifically, during the long interictal period, migraineurs display increased free water movement compared with controls in areas that process orofacial pain, such as the SpV, dlPons, and PAG/CNF. Remarkably, immediately before a migraine attack, this increase in diffusivity reduces to control levels before increasing again in the period immediately following migraine. It is clear from these data that in episodic migraineurs, regional brainstem microstructural changes occur throughout the migraine cycle, and that there are specific anatomical changes in the 24 h before onset.

A number of migraine studies have used DTI to identify anatomical changes in large fiber bundles such as the corpus callosum, internal capsule and corona radiata, although these studies did not explore changes in diffusion within gray matter or within fiber bundles in the brainstem (Neeb et al., 2015; Zhang et al., 2017). While a previous investigation used a region of interest approach to find that migraineurs show greater MD compared to controls within the red nucleus (Kara et al., 2013), no study has specifically explored the brainstem, particularly at different times over the migraine cycle. Consistent with a previous study, we found significant MD increases in SpV, dlPons and PAG/CNF (Marciszewski et al., 2018a), and furthermore, we show that during the 24-h period immediately before a migraine, these structural changes disappear so that migraineurs are no different from controls in the preictal period. However, these structural changes then return during the 72-h period following the migraine event. Furthermore, we found increased FA in the area encompassing the ascending VTT/VTA in migraineurs during all phases.

It has been argued by many that the headache period of migraine results from activation of trigeminal afferents in brain meninges and large cerebral arteries and these afferents terminate in the SpV and upper cervical dorsal horn (Liu et al., 2004, 2008; Olesen et al., 2009). While the nature of the cellular changes underlying such diffusion changes is unclear, MD changes can be associated with edema, vascular injury, inflammation, demyelination, cell count, and cellular morphology; as such our findings could therefore reflect several underlying biological changes (Alexander et al., 2007; Hutchinson et al., 2018). The dynamic nature of the changes reported here suggest that they reflect processes that are not permanent or static in nature, but that can instead change relatively rapidly, at least over the period of a day. Since MD changes can result from dynamic processes such as gliosis (Sierra et al., 2011), and there is evidence that migraine is associated with altered glial function (Nedergaard et al., 1995; Benarroch, 2005), it is possible that the MD decreases immediately before a migraine result from astrocyte activation and associated expansion of astrocytic processes. Indeed, a recent preclinical epilepsy investigation linked microstructural changes in astrocytic processes with altered measures of diffusivity (Salo et al., 2017).

Consistent with the idea that migraine is associated with astrocyte activation, it was recently shown that immediately before a migraine, resting infra-slow oscillatory activity (0.03–0.06 Hz) increases in these same brainstem regions (Meylakh et al., 2018). Astrocytes can exhibit infra-slow calcium oscillations that can propagate among surrounding astrocytes and it has been proposed that in pathologic situations, enhanced calcium-wave synchrony and amplitude may occur which can alter local neural function (Parri and Crunelli, 2001; Crunelli et al., 2002; Cunningham et al., 2006; Halassa et al., 2007; Lörincz et al., 2009). This raises the prospect that immediately before a migraine, astrocyte activation results in decreased MD and increased infra-slow oscillatory activity resulting in altered sensitivity within brainstem regions that receive and process orofacial noxious information. Whether such a sensitivity change is adequate to evoke head pain from basal levels of neural traffic or simply to facilitate an incoming trigger to activate higher brain centers to produce head pain remains to be determined.

The hypothesis that the brainstem pain processing sites become more sensitive as a migraine approaches was supported by Stankewitz and colleagues and Lee and colleagues, who reported that orofacial noxious and non-noxious stimuli evoked greater SpV signal intensity increases as a migraine attack approaches, although no significant change in the perceived intensity of orofacial stimuli (Stankewitz et al., 2011; Lee et al., 2017). Similarly, a recent study by Marciszewski and colleagues also reported that noxious stimuli evoked dramatic SpV activation increases, particularly in the 24-h period before a migraine (Marciszewski et al., 2018b). However, despite the increase in SpV activation, during acute orofacial stimuli, individuals’ reported pain intensity ratings decreased as a migraine approached. This appears at odds with the idea that brainstem pain-processing circuits become more sensitive as a migraine approaches although there are a number of potential explanations: (1) since these data reveal that both the anatomy and function of brainstem pain processing circuits are dynamic, it is possible that these pathways may change again at the onset or during a migraine itself; (2) since preclinical studies have shown convergence of dural-sensitive and facial cutaneous afferents in SpV (Burstein et al., 1998; Ellrich et al., 1999), a decrease in noxious cutaneous afferent drive onto second-order convergent SpV neurons may result in an overall increase in dural afferent input sensitivity; (3) changes in descending brainstem modulatory inputs onto the SpV may evoke a heightening of dural afferent input sensitivity at the expense of inputs from other orofacial structures. While these ideas are highly speculative, it is unlikely that the alterations in SpV anatomy and function are involved in other functions to the same degree as the processing of orofacial noxious afferents.

While our data imply that the processes involved in migraine attack onset may be astrocytic in nature, whether astrocyte activity is specifically driving migraine initiation or simply a symptom of another process cannot currently be discerned. The gradual increase in MD in brainstem pain-related regions over the interictal period suggests that changes are occurring throughout the interictal period that then dramatically reverse immediately before a migraine to control levels. While brainstem functional measures such as SpV signal intensity changes and infra-slow oscillations were at control levels during the interictal period and dramatically increased immediately before a migraine, MD was above control levels during the interictal period and reduced to control levels before migraine. This implies that brainstem anatomy is not simply changing before a migraine but is altered throughout the long interictal period.

Several reports suggest reduced endogenous analgesic ability in migraine (Sandrini et al., 2006; de Tommaso et al., 2007) while others report no change (Perrotta et al., 2010; Teepker et al., 2014). This inconsistency may reflect subtle variations in endogenous analgesic responsiveness across the migraine cycle, and it might be that endogenous analgesic ability gradually increases over the interictal period which is consistent with MD increases in pain-processing and modulating regions across the interictal period. Additionally, none of the regional microstructural changes we detected were correlated to migraine properties such as migraine frequency, intensity or duration, suggesting that the changes are not cumulative over time, and is consistent with the idea that they may be dynamic in nature. While these results are in line with some migraine studies (Chen et al., 2016; Uggetti et al., 2017), others have reported significant linear relationships between anatomical measures and migraine frequency (Kruit et al., 2009; Mainero et al., 2011), intensity (Russo et al., 2012), and years suffering (Liu et al., 2012; Schwedt et al., 2013; Rocca et al., 2014; Chong and Schwedt, 2015); however, none of these studies explored the brainstem.

In addition to diffusion changes associated with brainstem nuclei, we found FA increases in migraneurs that encompassed the VTT/VTA. These FA increases were not dynamic and occurred at all migraine phases, indicating a constant underlying change, possibly in the ascending trigeminal pain pathway from SpV to the thalamus. While it has been suggested that altered radial diffusion within a fiber tract may represent increases in response to demyelination (Song et al., 2005) and axial diffusivity to axonal damage (Song et al., 2003; Loy et al., 2007), we found no change in axial or radial diffusivity associated with the increase in FA; although it is unclear how accurately the directional diffusivities relate to specific pathologies. Furthermore, while we found primarily changes in axial and not radial diffusivity in brainstem nuclei, what these changes represent remains unknown, although it is not inconsistent with altered astrocyte activation.

There are a number of methodological and subject-related limitations of this study. The spatial resolution of human DTI is relatively low and thus it is difficult to precisely localize each brainstem cluster with respect to small brainstem nuclei. However, the location of each cluster was defined using brainstem atlases and we placed the changes into context with respect to the existing human and preclinical research. Secondly, although we managed to scan 13 migraineurs immediately before a migraine, only seven migraineurs were scanned during all three phases and we did not scan migraineurs during a migraine attack itself. However, we did use population-based statistics with thresholds corrected for multiple comparisons, and although a larger subject number is always preferable, we are confident our results are robust. Future studies in which the migraine phase itself was also explored would further increase our understanding of anatomical changes across the migraine cycle. Thirdly, this is a largely cross-sectional study and with mounting evidence of relatively rapid brainstem changes, scanning individual migraineurs over the course of several weeks while measuring indices of brain anatomy, activity, and sensitivity would provide more precise evidence supporting this hypothesis. Furthermore, although the diffusivity changes reported here are dynamic in nature, i.e., over a period of days, it remains unknown if such changes are dynamic enough to be altered by transient activation of cell populations.

Overall, our findings suggest that migraine is associated with anatomical changes within brainstem structures involved in trigeminal noxious transmission and endogenous analgesia. More importantly, these anatomical changes alter over the migraine cycle specifically during the 24-h period before a migraine attack. We speculate that these anatomical changes reflect astrocyte activation that alters local neural function by the release of gliotransmitters, which either trigger or alter the sensitivity of the brainstem so that an external trigger induces a migraine attack. Future investigations exploring brainstem resting activity, evoked activity and anatomy over the migraine cycle may provide evidence supporting such a proposal. If dynamic changes in brainstem function and structure do occur, we may be in a position to modify these changes and potentially prevent the triggering of a migraine attack.

## Synthesis

Reviewing Editor: Karen Davis, Krembil Research Institute and University of Toronto Decisions are customarily a result of the Reviewing Editor and the peer reviewers coming together and discussing their recommendations until a consensus is reached. When revisions are invited, a fact-based synthesis statement explaining their decision and outlining what is needed to prepare a revision will be listed below. The following reviewer(s) agreed to reveal their identity: Mojgan Hodaie, Vitaly Napadow.

The authors have addressed the comments by the reviewers and the manuscript is much improved.

The issue of imaging of migraneurs and timing with respect to the onset of migraine will always remain an issue, however it is important to strive towards some form of a comparative group. The authors should take this into consideration for future studies, as it will strengthen their results.

There are just a few minor comments to address:

1. For many of the significant clusters shown in the brainstem, the gray matter (nucleus) is localized and named. However, in the midbrain, the authors do not note VTA - ventral tegmental area - but instead note ventral TT, a white matter bundle/tract. Perhaps some consistency is warranted here, unless we're focused on a skeletonized analysis, which is not the case in the brainstem, the significant “clusters” could refer to either nuclei or ascending/descending white matter tracts and should be labeled consistently, unless there is good rationale to not do so.

2. In Table 1, cluster size should note units. Is this number of voxels? Volume in mm^3 may be better, but either way, I would note the units here.
